# Eudaimonia and well-being: questioning the moral authority of advance directives in dementia

**DOI:** 10.1007/s11017-020-09517-w

**Published:** 2020-02-07

**Authors:** Philippa Byers

**Affiliations:** Plunkett Centre for Ethics, St Vincent’s Hospital Sydney and Australian Catholic University, Darlinghurst, NSW Australia

**Keywords:** Dementia, Advance directives, Eudaimonia, Well-being, Ronald Dworkin

## Abstract

This paper revisits Ronald Dworkin’s influential position that a person’s advance directive for future health care and medical treatment retains its moral authority beyond the onset of dementia, even when respecting this authority involves foreshortening the life of someone who is happy and content and who no longer remembers or identifies with instructions included within the advance directive. The analysis distils a eudaimonist perspective from Dworkin’s argument and traces variations of this perspective in further arguments for the moral authority of advance directives by other authors. It then critiques a feature of the eudaimonist perspectives within these arguments—namely, the position that dementia has a retroactive negative impact on what a person has previously valued—and challenges the commonly held assumption underlying them that a person’s life and well-being have relatively low value beyond the onset of dementia. Although advance directives have moral authority as a means of guiding one’s future health care, accounts that dismiss the value of the lives and well-being of people living with dementia should be questioned to the extent that such accounts are used to support the moral authority of advance directives stipulating measures to foreshorten individuals’ lives.

## Introduction

An advance directive (AD) is a set of written instructions that sets out preferences and guidance for future health care and medical treatment in the event that a person is unable to direct her or his own care or treatment due to a medical emergency or to advancing illness. ADs can include requests that no treatment be given or that treatment be withdrawn in certain circumstances, reflecting the right of decisionally competent patients to refuse treatment. However, there is an ongoing debate over whether advance directives retain their moral authority beyond dementia onset when decisional competence is impaired. The ADs most at issue in this debate are those that stipulate nontreatment or treatment withdrawal with the express purpose of foreshortening a person’s life after dementia onset.

The AD debate raises questions on a range of issues, such as (1) the authority of ADs in light of possible discontinuities in a person’s identity beyond dementia onset, (2) differences in the scope or limits of autonomy before and after the onset of dementia, and (3) the extent to which one’s previous preferences, desires, values, and opinions should be the most decisive factor in determining one’s best interests after dementia onset.

Legal philosopher and rights theorist Ronald Dworkin set the original terms for the ongoing AD debate [1, pp. 218–237; 2]. His positions on the above three issues are as follows: he believes (1) that there is continuity in personal identity beyond dementia onset, such that individuals’ prior written ADs apply at a later time as the authoritative expression of their preferences and instructions even if they no longer remember or identify with the underlying reasons for their former preferences and instruction; (2) that the autonomy expressed through an AD extends beyond the memory loss that may result from dementia onset, and said autonomy should not be limited by other considerations such as individuals’ current well-being or their apparent desire to continue living; and (3) that one’s prior preferences, desires, values, and opinions are decisive for determining one’s best interests after dementia onset, although adherence to them may involve foreshortening life at a time when one has no wish to die.

Dworkin sharpens his position on issues (2) and (3) by appealing to the case of Margo. Margo was a middle-aged woman with advancing dementia who was very sociable, was clearly enjoying her life, and expressed no wish to die. Dworkin’s position is that even for persons with dementia who may be as happy and contented as Margo, a previously written dementia-specific AD should be enacted to foreshorten their lives, since upholding the AD is in their best interests irrespective of their current well-being. The implication is that the well-being of a person is not a relevant factor in determining the moral authority of ADs that stipulate life-limiting measures after the onset of dementia.

Rebecca Dresser and Agnieszka Jaworska have proposed what are now well-known arguments against Dworkin’s position. Dresser argues that dementia-specific ADs should not be regarded as well-informed expressions of a person’s will after the onset of dementia, given that prior to dementia onset it is not possible to know how one’s values and appreciation of life may change in that event [3, 4].^1^ Jaworska disputes Dworkin’s characterisation of a person’s interests after dementia onset as simply ‘in the moment’, arguing that one’s former capacities for valuing can remain largely intact despite cognitive loss [5]. These arguments cast doubt on Dworkin’s view that one’s current desires and interests have little moral purchase, in their own right, once one is living with dementia.

More recently, Jennifer Hawkins [7] has focussed on issue (3). She argues that although there may be other arguments for the moral authority of dementia-specific ADs, it is not tenable to believe that enacting an AD is in the best interests of a person with dementia who is nonetheless happy and content and who no longer remembers or identifies with the instructions and preferences within her AD. Hawkins proposes a basic or minimum principle of well-being which she believes has general application—which is to say, applies irrespective of whether a person has dementia. According to this principle, which she calls the *non*-*alienness* principle, whatever is deemed to be good for a person or conducive to her well-being, and thereby determined to be in her best interests, must have a positive impact on that person’s current experience or be positively evaluated by her at the time that it is deemed to be good for her. On this basis, Hawkins argues that when individuals with dementia are happy and content and no longer remember or identify with the preferences and instructions within their ADs, enacting a dementia-specific AD is simply not good for them and thus is not in their best interests. Hawkins’s position is that criteria for determining individuals’ best interests should apply contemporaneously to any action done for the sake of their best interests, including the enactment of ADs.

This paper will focus on a eudaimonist perspective, as I refer to it, that is present within prominent arguments for the moral authority of ADs in dementia. It will highlight and then challenge two implications of this perspective: (1) that dementia has a retroactive negative impact on life projects or the value of a person’s life as a whole, and (2) that the short-term well-being of those living with dementia has little moral value over and above a simple welfare value and, as such, makes very little to no contribution to their current lives or to their lives as conceived from a former perspective.

## Defining a eudaimonist perspective

The term ‘well-being’ is typically seen to have two senses. The first is *short*-*term well*-*being* (STWB) as a sense of one’s presently experienced state of contentment and happiness, taking account of individuals’ desires and interests as conceived from their current perspective. The second is *long*-*term well*-*being* (LTWB) as an overarching sense of one’s lifetime happiness and fulfilment.

Within discussions of ethics, LTWB is often referred to as *eudaimonia*, an ancient Greek term that can be translated as happiness or flourishing across a lifetime. Although STWB and LTWB are interrelated in complex ways, LTWB is sometimes distinguished from STWB and accorded greater value or significance. For example, a person may decide to put his STWB on hold for the sake of his LTWB, say, by studying something dull over the holidays rather than relaxing with friends, doing so for the sake of a lifetime goal that will contribute to LTWB. The prioritisation of long-term over short-term well-being seems to be a characteristic feature of prudential reasoning and a characteristic feature of approaching one’s own life as an ethical, aesthetic, or narrative project. Aristotle’s thinking is along these lines, considering the central question of ethics to be how to live the best life for a human being and, as such, holding the primary purpose of ethics to be the achievement of eudaimonia, as a state of happiness or flourishing across a lifetime [8].

In the following section, I draw out a eudaimonist perspective present within Dworkin’s argument for the position that ADs have moral authority after the onset of dementia, which I then link to three other arguments for the same position made by Norman Cantor [9], Jeff McMahan [10], and David DeGrazia [11], respectively. In broad terms, what I mean by a eudaimonist perspective is a view that accords moral priority to the personal and individual task of giving shape to either one’s life conceived as a whole, or one’s personality, or one’s long-term interests or conception of identity—over and above considerations of short-term well-being. My purpose is not to critique eudaimonist views or perspectives as such, as I dispute neither that giving shape to one’s life, personality, interests, and conception of identity is a worthy goal, nor that living well often requires trade-offs between short-term happiness and longer-term projects, nor that the autonomy that life-shaping presupposes and requires is morally insignificant. Rather, my purpose is to seriously question the low value accorded to the experiences, desires, and interests of persons living with dementia—and thus to their STWB—when eudaimonist perspectives are advanced within pro-autonomy arguments for the moral authority of ADs that stipulate life-limiting measures after the onset of dementia.^2^

## Four contemporary eudaimonist perspectives

Dworkin believes that ADs are an important means of ensuring that individuals’ lives can end in a manner that is consistent with the coherence and integrity of their lives as a whole, if such a consideration is important to them prior to dementia onset. Rather than explicitly discussing values, Dworkin describes two forms of interests he refers to as critical and experiential interests [2, pp. 201–202]. Both forms of interests can be thought of as desires or intentions, as both have objects or goals. As the name suggests, *experiential interests* are realised or satisfied through experiences, and thus may be short-lived and intermittent—for example, the pleasure of eating when hungry or the pleasing warmth of a heater on a cold day. If considered independently of other interests, experiential interests are relatively simple, short-lived, or time-bound.

In contrast, *critical interests* are not realised or satisfied through specific experiences, in the sense of being fully satisfied or realised at particular—which is to say, potentially specifiable—times. Critical interests include the self-made plans that give overall shape and meaning to a person’s life. For Dworkin, a legitimate object of a critical interest can be one’s life considered as a whole, or as an entity that is distinguishable from the moment-by-moment experiential processes involved in living and distinct from the sum of all of one’s particular life experiences. Importantly, a critical interest can extend beyond the time it is expressed or actively endorsed and can be realised independently of the experiential perspective of the person who has adopted it.

Dworkin draws a sharp, evaluative distinction between critical and experiential interests—that is, a distinction between one’s interest in the coherence and integrity of one’s life as a whole, which can include an interest in how it ends, and one’s interest in feeling good, which consists in moment-by-moment experiences. With this sharp distinction in place, Dworkin also characterises persons with dementia as having experiential interests only [2, pp. 229–230], and thus as having limited, moment-by-moment interests that can be relatively easily satisfied and have little value over and above a simple welfare value.^3^ Accordingly, persons living with dementia, and the lives that they lead, are presented as making little to no contribution to their lives considered as a whole from an earlier eudaimonist perspective; and those lives—the ones considered as a whole from the earlier perspective—are seen to have moral priority over the lives that persons with dementia are currently leading.^4^

While Dworkin argues for the moral authority of ‘no treatment’ stipulations in ADs, such that an infection might be left untreated as a means to limit or foreshorten life, Norman Cantor appeals to a similar eudaimonist perspective to argue for a more pre-emptive approach [9]. He notes that the middle stage of dementia can last quite a while, and during this time there may not be an infection or other call for life-saving intervention, and hence there may not be an opportunity to enact a ‘no treatment’ stipulation within a dementia-specific AD. He considers legal precedent in the United States that might enable patients to stipulate that no food or hydration be offered past a certain point of dementia progression, which is a point of reduced cognitive functioning that people should be able to identify for themselves and set out within their own ADs. Cantor then carefully describes how pain management and sedation could be administered to avoid pain and discomfort after the withdrawal of food and hydration. His goal is to extend the range of what can be withdrawn or implemented, thus extending the possible ways in which the life of a person with dementia could be foreshortened if so desired prior to the onset of dementia. And while he acknowledges a moral obligation, should he develop dementia, not to cause pain and distress to his future self on the basis of welfare considerations, he (like Dworkin) accords little value to the STWB of that future person over and above a simple welfare value.

Cantor argues that living with dementia, contentedly or otherwise, is at odds with his ‘personal vision of dignity’, and he is disposed to ‘care mightily about posthumous recollections of [his] personality’, which is a personality he has deliberately cultivated [9, p. 16]. He argues that living with dementia would be at odds with, or a threat to, what he strongly values in himself both now and in prospect.^5^

Dworkin and Cantor each believes that dementia involves a loss of, or at least a serious compromise of, certain goods that they both value. In Dworkin’s argument, this good is a life considered as a whole with an ending that aligns with its former self-directed shape. This is analogous to a narrative ending, as Dworkin notes [2, pp. 210–211]. A well-crafted narrative requires a well-crafted end for its meaning to be captured in its entirety. In terms of achieving full or rich meanings in narratives, certain endings are better or worse vis-à-vis the expectations of the genre. Similarly, in terms of actualising the self-directed meanings of lives, certain endings are better or worse vis-à-vis the ideals of the individual, at least insofar as she or he retains the capacity to hold the end of life in view, so to speak. In Cantor’s argument, the good he strongly values, and believes that dementia threatens, is a personality realised in traits and capacities that are deliberately cultivated—a personality that he hopes will endure both during his lifetime and beyond it in the form of predominant memories in others.

A life as a completed whole (in Dworkin’s sense) and the actual endurance of a specific personality in memory (as Cantor envisages) are not goods that can be attained or known about on the basis of one’s own experience. As such, they are both non-experiential goods. Accordingly, both authors are arguing that individuals’ interests in these non-experiential goods should be respected both before dementia onset and beyond it, to a time when they may no longer value the same goods and their desires and interests may have changed. The clear implication is that the STWB of a later self with dementia is of low value and can be discounted for the sake of the non-experiential goods and eudaimonist conceptions of a well-crafted life or personality, which each author values more highly.

Dworkin and Cantor both assume that there is continuity in a person’s identity after the onset of dementia, holding that the moral authority of someone’s AD prior to dementia extends beyond dementia onset as it applies to the same person. But the use of a eudaimonist perspective in support of the moral authority of ADs in dementia seems not to require the assumption of a continuous identity through the onset of dementia. Jeff McMahan and David DeGrazia both adopt a eudaimonist perspective as part of their arguments for the moral authority of ADs in dementia [10, 11], drawing on an approach to identity originally developed by Derek Parfit whereby what matters in terms of future-directed concern is continuity of psychological relations, rather than the numerical identity of a continuing, embodied person [14, 15].

McMahan provides a minimalist account of personal identity which forms the basis of an account of interests that can be morally evaluated in utilitarian terms, both interpersonally and intrapersonally. The account of identity is as follows. McMahan suggests there needs to be enough of a functioning brain to support or realise ‘consciousness or mental activity’ [10, pp. 66–68]. He says:

I believe that there need be only enough physical and functional continuity [of the brain] to preserve certain basic psychological capacities, particularly the capacity for consciousness. This, I believe, is a sufficient basis for egoistic concern; it should, therefore, be a sufficient basis for identity, other things being equal. [10, p. 69]

Although the account seems sparse, in the sense that ‘physical and functional continuity’ of the brain is of only derivative significance to the capacity for consciousness that the brain supports, this position provides McMahan with a basis for arguing ‘that we are essentially embodied *minds*’ [10, p. 68].

Despite McMahan’s claim that we are embodied minds, and his view that numerical identity and singular embodiment coincide with one another, McMahan also believes that who we are as persons can trail off by degrees or be ‘indeterminate’ [10, p. 44]. The indeterminacy, or trailing off, of personhood is the result of weakening psychological relations and diminishing physical and functional continuity of the brain. This claim about indeterminacy is strongly resonant of Parfit’s approach and underpins McMahan’s view of personhood beyond the onset of dementia, which lends support to his view that its value is low.

For McMahan, the degree or extent to which psychological relations are either weak or strong depends on two factors: (1) the internal unity and richness of individuals’ psychological relations in conjunction with (2) the temporal reach of their psychological relations. The internal unity and temporal reach of psychological relations give rise to what he calls *time*-*relative interests*, which he suggests can hold by degrees. McMahan’s objective is to provide disinterested, moral answers to so-called ‘hard cases’ in bioethics by accounting for the ways that different sets of interests can be ranked relative to one another.^6^ In brief, his view is that a person’s interests count more or less, morally speaking, depending on the extent or degree to which they are time-relative. He argues that in order to have interests at all, one must have sentience and conscious awareness. Beyond this pre-condition for moral consideration, there are differing degrees of time-relative interests, which increase with progressive levels of development or decrease with significant cognitive impairment.

McMahan suggests that at early stages of dementia, persons are fully themselves, given that the internal unity and temporal reach of their psychological relations are largely unaffected while episodic memories are still intact. But at later stages of dementia, there is no longer the same internal unity or temporal reach of psychological relations, leading McMahan to conclude that a person with late-stage dementia is ‘barely there at all’ [10, p. 494]. McMahan also suggests that at early stages of dementia, a person should have little prudential concern for a future ‘barely-there’ person with late-stage dementia, as the temporal reach of the psychological relations between them will be weak. This is also Parfit’s view—that prudential concern should track the connectedness and continuity of psychological relations rather than the numerical identity of an embodied living person.

McMahan believes his account provides a disinterested means of morally evaluating the here-and-now interests of persons with late-stage dementia relative to time-relative interests of persons at an earlier stage of life. In his view, these sets of interests are almost entirely separate as they are only weakly connected to one another. He also believes that respecting a person requires respecting her time-relative interests as they were prior to dementia onset or at a very early stage of dementia. The interests of a ‘barely-there’ person at a later stage of dementia matter much less, and the STWB of such a person at that time counts for very little in a supposedly morally disinterested consideration of interests.^7^

David DeGrazia’s views are also indebted to Parfit’s approach to identity. His overarching position on who or what we are holds that we are human animals that have a narrative sense of identity [11]. He suggests that our numerical identity is biological, since our persistence as single continuing individuals depends on embodied, biological continuity. However, he distinguishes the numerical identity that accompanies biological embodiment from a psychological notion of identification, arguing that what we identify with, and thus what matters to us, is a narratively constituted sense of self-identity, or narrative sense identity for short, which is crucially intertwined with our values [11, pp. 200–201]. In ordinary cases, narrative identity coincides with and presupposes the numerical identity, or persistence, of our singular animal embodiment. But DeGrazia believes that identification with a narratively constituted sense of self can be considered separately and understood as mattering to us in a very different way.^8^ This particular view brings out his alignment with Parfit: a prudential concern with interests—as that which we seek, or care about, or identify with—can be separated from the numerical identity of the embodied living person we ordinarily take ourselves to be.

DeGrazia adopts Marya Schechtman’s approach to identity and self-constitution [16], proposing that narrative identity is what is threatened when one has an ‘identity crisis’ and must grapple with questions such as ‘Who am I really?’ [11, p. 83]. This sense of identity is at stake in matters of future-directed prudential concern—as, for example, when individuals wonder what will become of them as they consider different life options in their early lives. Hence this narrative sense of identity involves the possibility of self-creation—of becoming, or having been, a person whose life is shaped by a particular view of ideals and values. DeGrazia suggests that this narrative sense of selfhood or identity, and the threats and possibilities it involves, is dependent on a complex set of conscious capacities such that it is diminished if these capacities are impaired.

DeGrazia notes that this narrative sense of identity is reminiscent of McMahan’s views on the internal unity and temporal reach of psychological relations, but he suggests that it both provides a better account of what matters to us as we reflect on our identities and has a stronger bearing on the intuition that dementia-specific ADs are morally authoritative beyond the onset of dementia. Like Dworkin, DeGrazia believes that the end of life is analogous to the end of a narrative in the sense that the quality of a narrative ending affects the meaning (or value) of what comes before it.^9^ To take the analogy between narratives and lives in a semi-literal way is to imply that an unchosen or unwished-for ending compromises the overall quality of the life that precedes it, which is a view with high stakes when making value judgments about the lives and well-being of people with dementia.

## A retroactive negative impact on value?

Cantor insists that dementia would ‘soil’ the image of his personality as he himself has cultivated it, an image that he highly values, regarding dementia as detracting from and having a retroactive negative impact on this value [9, p. 16]. McMahan believes that a period of life with dementia is ‘an excrescence dangling at the end [of a life]—a period alien to and … distinct from the earlier unified life’ [10, p. 501]. And although McMahan believes that a later person with dementia is ‘barely there’, he also holds that the continued existence of that person ‘can retroactively affect the meaning and value of her life prior to the onset of dementia’ [10, p. 502].^10^ In this way, Cantor and McMahan both believe that dementia has the effect of spoiling what comes before it; for them, it is simply the case that a personality, or set of psychologically connected interests, is compromised by the cognitive impairments entailed by dementia. DeGrazia also suggests that a protracted ending to a life with dementia has the effect of spoiling that life insofar as the individual in question has previously anticipated dementia to offer a ‘degraded existence’ [11, p. 194]. On this point, I note that although Dworkin’s argument is the first in a series of arguments for the moral authority of dementia-specific ADs which incorporate the eudaimonist perspective treated in this paper, Dworkin does not articulate a view about the retroactive impact of dementia on the value of a person’s life in the same strongly negative terms as Cantor, McMahan, and DeGrazia.

Despite their difference in emphasis, the four authors share the following views: (1) the value of the STWB of a person living with dementia is low and (2) having dementia at the end of life has a retroactive negative impact on some good that one has previously valued. For Dworkin, this good is a life conceived as a unified whole up to and including its end, akin to the end of a unified narrative, uncompromised by the progression of dementia. For Cantor, this good is a cultivated personality that endures unsoiled by dementia, both in the course of one’s life and beyond it in the memory of others. For McMahan, this good is the unified set of psychological interests and relations of a ‘fully-there’ person, the value of which has not been retroactively compromised by the weakly connected psychological relations of a ‘barely-there’ person with dementia. For DeGrazia, this good is a narratively constituted sense of identity, as conceived and identified with by a person for whom dementia represents a spoiling of that identity.

In each case, the STWB of a person with dementia is accorded little value other than a welfare value, which serves only as a check against causing or allowing a person with dementia to suffer preventable pain or discomfort. The STWB of a person with dementia is assumed neither to have current value to the person who is living with dementia nor to contribute any value to that person’s life in a longer-term sense. Rather, the continuing lives of persons with dementia, and a fortiori their STWB, are seen as undermining the value of their whole lives, or personalities, or sets of psychological interests and relations, or narratively constituted identities.

I suggest questioning the view that foreshortening the life of a person with dementia is a means of retaining or preserving what is seen as having value from a former eudaimonist perspective. To flesh this out, I will turn to David Velleman’s discussion of the respective values of STWB and LTWB [20].

Velleman proposes that retrospective significance—such as when one views an event differently due to a change in circumstances or outlook—does not affect the value of the event as it was conceived at the time. I believe this is correct, both in general and in the case of dementia. I also believe Velleman is correct when he suggests there is a *second*-*order good* that derives from a particular temporal distribution whereby earlier goods contribute to the value of later ones, as is the case when a person’s life is going well such that he can be described as having LTWB.

When a person’s life is seen to be going well, there may be a discernible forward-going direction of significance, with earlier actions and events contributing ongoing significance to that person’s life. For example, a person who has made good use of opportunities may find she is well-placed to give a further, ongoing shape to her life, drawing on past experiences as she does so. As individuals develop wisdom and make good use of the opportunities they encounter and the skills they cultivate, perhaps overcoming adversity along the way, the benefits of life experience and wisdom accrue incrementally.

A life with an uphill trajectory, where adversity is overcome and success accrues later in life rather than earlier, is typically seen as preferable to a life with a downhill trajectory, where ease and success early in life give way to hardship later on.^11^ The preference stands even if one imagines two cases where the amount of time with STWB is greater within a lifetime with a downhill trajectory than within a lifetime with an uphill trajectory. This general preference for the temporal distribution of well-being that goes with an uphill life trajectory seems to indicate that LTWB is a distinctive eudaimonist good that is independent of a person’s STWB, but I believe this conclusion is too strong.

The four authors discussed above believe a decline in good fortune due to the onset of dementia compromises value—that ending life with dementia compromises the value of that life as a whole, or compromises the value of a personality, and so forth. Ending life with dementia not only produces a downhill trajectory, but also detracts from the value of whatever is the case prior to dementia onset. Accordingly, enacting an AD is seen as a means of retaining or preserving that which is previously deemed to have value. I dispute this implication of the eudaimonist perspective—namely, that enacting an AD retains or preserves the value of something as it was prior to dementia onset where dementia is seen to soil or spoil this value. I suggest that a change in life circumstances due to the onset of dementia undermines neither the second-order value of the LTWB that came before it nor the value of other things people deem to have long-term significance prior to dementia.

## Dementia as a moral loss?

The onset of dementia is unbidden and unwelcome, but I suggest it does not have the retroactive negative impact on value that Dworkin, Cantor, McMahan, and DeGrazia assume. Depending on their level of insight as dementia progresses, individuals may experience periods that are difficult and unpleasant given that they are aware of oncoming cognitive impairment, which of course is undesired.^12^ The onset of dementia also means that a person’s life will end differently than it might have ended absent progressive cognitive impairment. In this sense, the onset of dementia represents the loss of a future that may have been. But the onset of dementia does not undermine the value of what has been. There are moral losses that do have this kind of retroactive negative impact on value, but I propose that dementia is not one of them.

A retroactive negative impact on value can occur when a person discovers that moral assumptions about a long-term life partner are incorrect—for example, when it comes to light that a life partner has always had a secret and morally duplicitous double life that is entirely at odds with her or his former moral self-presentation. This is a discovery that undermines the value of the shared life prior to the unwelcome discovery. It is a moral loss, a loss of the moral value the shared life was deemed to have, and it undermines future prospects as they were previously envisaged.^13^ As mentioned above, Velleman suggests that retrospective significance—such as when one views an event differently due to a change in circumstances or outlook—does not affect the value of the event as it was conceived at the time. I believe this to be so in most instances and certainly in cases of dementia. The exceptions are moral losses of the kind described above—losses of what a person thought was the case when the moral untruth or lower worth of what was actually the case comes to light.

The onset and progression of dementia bring losses, but these are not necessarily moral losses that have a retroactive negative impact on what was previously valuable.^14^ A previously well-lived life has been valuable and is not made less valuable by the onset of dementia, no matter how unwelcome that onset may be. The onset of dementia does mean that life will change, and some capacities and capabilities will be impaired. But these unwelcome changes and impairments do not entail a loss of the moral value of what has been the case, nor do they entail that the STWB of a person living with dementia has little value in and of itself. A person’s well-being is valuable from her or his own perspective, which typically continues to be an evaluative perspective despite the onset and progression of dementia, as argued by Jaworska and Sabat [5, 13]. Likewise, depending on individual circumstances and outlooks, a person’s well-being can be valuable from the perspective of those who may nonetheless wish that their loved one had not developed dementia. A person’s well-being is also valuable from the perspective of those working in dementia care that is person-centred and focussed on the needs and dignity requirements of those who are living with dementia, and sometimes living quite well despite their memory loss and cognitive impairment.^15^ Tia Powell suggests:

A life with dementia will always contain much that is hard, but it can also include more moments of joy. We’d have to see dementia, and people with dementia, and even perhaps ourselves with dementia, as needing what we all want: peace, comfort, companionship, care. [26, p. 74]

Seeing the ‘peace, comfort, companionship, care’ of people living with dementia as valuable in its own right is directly connected to seeing their STWB as having clear and unequivocal value. Regarding the STWB of people with dementia as having clear and unequivocal value is connected to regarding STWB—of persons with or without dementia—as having value in its own right, as Velleman proposes. This view stands in sharp contrast to the eudaimonist perspectives of Dworkin and others.

Velleman suggests that ‘a person’s well-being has both a synchronic and a diachronic dimension’, maintaining that a person’s well-being in the latter diachronic sense (LTWB) is not reducible to ‘how well off he is at each moment’ [20, p. 158]. On this basis, he observes that LTWB has a second-order value. He then posits that we typically occupy two kinds of temporal perspectives: (1) momentary or synchronic perspectives, which are successive, and (2) overarching diachronic perspectives, which are accessed via successive perspectives. On Velleman’s view, what is good for a person from one of these perspectives is also good for a person from the other. In the same way, what is valuable to a person from one kind of perspective is also valuable to a person from the other. It is worth noting that Velleman does not consider whether the current perspective and STWB of a person living with dementia add value to a long-term perspective. I am not arguing that short-term perspectives always add value; rather, I am problematising the assumption that living with dementia could never add value to a person’s life as well as the assumption that dementia somehow lessens or detracts from what was valued from a former long-term standpoint.

In Velleman’s view, the value of moment-by-moment experience is not solely derived from the value of LTWB; and, relatedly, the diachronic perspective on one’s life as a whole is not more authoritative than synchronic perspectives:

My brief is on behalf of all momentary perspectives equally, against the assumption that their deliverances are to be overridden by those of the diachronic perspective that subsumes them. … The good that something does you now is not just the phantom of a restricted method of accounting; it’s an autonomous mode of value. [20, p. 169]

Velleman argues that STWB has its own value, and according a distinctive or second-order value to LTWB need not involve according a relatively lower value to STWB. Returning to the issue of dementia, given that STWB has a value that is not solely derived from its contribution to LTWB, the STWB of a person with dementia should not be discounted or summarily dismissed in arguments for the moral authority of ADs. This is Jennifer Hawkins’s position [7].

Hawkins suggests that individuals’ previous or longer-term desires can play an important epistemic role when determining what conduces to their current STWB. For example, if a person has often enjoyed listening to classical music in the past, it is likely—though not guaranteed—that he will derive pleasure from listening to classical music now and in the future.^16^ Despite the potential epistemic role of past desires, Hawkins questions whether past preferences and desires should be the decisive moral considerations when determining a person’s current best interests, particularly the preferences and desires that are included in previously written ADs but play no role in individuals’ subsequent perspectives or outlooks as they no longer remember or identify with those preferences or desires. As described above, Hawkins proposes a non-alienness principle of well-being, which prioritises the current experiences and evaluative stance of any person for whom a question of well-being arises, irrespective of dementia.

Hawkins’s non-alienness principle allows for changes in mind, circumstances, capacities, and outlook—changes that Dresser argues cannot be reliably foreseen prior to dementia onset, irrespective of how unwelcome the prospect of dementia may be. I do not argue that the current interests of individuals living with dementia must always be prioritised over and above their previous preferences and desires. Rather, I propose that when moral priority and authority are considered, it should not be assumed in advance that the life, evaluative perspective, and STWB of those living with dementia are of low value. And one should not assume that the STWB of persons living with dementia has nothing whatsoever to contribute to their LTWB—or what could be one’s own LTWB in the future.

## Conclusion

Individuals’ short-term well-being is valuable in its own right and a morally relevant factor in decisions that concern them, irrespective of whether they are living with dementia. Refining current understandings of what well-being in dementia involves, and how it may be lifted and supported in spite of progressive cognitive impairment, is not necessarily a simple matter. But given the scale of dementia incidence in our communities, I suggest that giving due moral consideration to the well-being of people living with dementia is a pressing contemporary issue. Arguments that do not accord value to the well-being of people living with dementia, over and above a simple welfare value, strike me as far too dismissive of individuals’ lives, concerns, and evaluative perspectives beyond the onset of dementia.

I believe there is reason to question whether valuing well-crafted lives, cultivated personalities, temporally extended and unified interests, and narratively constituted identities is bolstered by undervaluing the well-being and lives of persons with dementia. One can value these longer-term things without making a correlative assumption that the well-being and lives of those who currently live with dementia, or will live with it in future, have a low value. This value assumption should be problematised especially when it is put forward as support for the moral authority of measures that are intended to foreshorten individuals’ lives. Ultimately, any form of valuing and correlative disvaluing should be questioned when it serves to heighten the vulnerability of those who are already vulnerable.

## Footnotes

1 David Shaw has argued for a similar view, which he situates with reference to legislation within the United Kingdom [6].

2 This paper addresses the role of eudaimonist perspectives in moral justifications for enacting dementia-specific ADs; it does not address eudaimonist justifications for suicide or for the assisted death of decisionally competent patients (cf. [12]).

3 As mentioned above, Jaworska has a very different view, which is echoed in Steven Sabat’s extensive research on people living with dementia. His research highlights the meaningfulness of their experiences from their own perspective, which is often overlooked or dismissed [13].

4 In characterising persons living with dementia as having experiential interests only, Dworkin rules out the possibility that they could have critical interests. Dworkin’s position is not that critical interests do not ever change, as a reviewer notes, but that persons living with dementia simply have no critical interests. If it is granted that a person’s well-being after dementia onset has worth, it is harder to grant that it is never the case, or that it simply could not be the case, that those living with dementia have critical interests that differ from their earlier ones.

5 Cantor would also like to ensure that his family are not burdened with his care, even if they would happily accept the responsibility. As a reviewer notes, this can be considered an altruistic justification that may add weight to the moral authority of an AD.

6 McMahan applies utilitarian reasoning to what he says are marginal cases in bioethics, but suggests that in day-to-day cases equal respect for persons applies and the same utilitarian approach is inapplicable.

7 McMahan writes, ‘the preferences of the demented are notoriously arbitrary, whimsical, and ephemeral’ and such individuals are capable of living only in ‘a condition of bovine contentment’ [10, pp. 497–498, 500]. As mentioned in footnote 3 above, this view is at odds with Jaworska’s argument and Sabat’s research [5, 13].

8 DeGrazia believes narrative identity and numerical identity can come apart in brain transplantation cases [11, pp. 75–76], which he regards as feasible and thus worthy of consideration.

9 Paul Ricoeur suggests that the analogy between lives and stories has limits, as ‘stories are recounted and not lived; life is lived and not recounted’ [17, p. 20].

10 Patrick Delaere also discusses the passage these remarks are drawn from [18]. Delaere focusses on the point that, according to McMahan, it would be better for a person with dementia to be treated, rather than have her life foreshortened in accordance with a dementia-specific AD, *except* for the fact that her life has a dependency relation on a former more unified life, with her continued existence negatively impacting on the unity of that life. Put differently, were it not for this dependency relation, having later-stage dementia would have no impact on the unity of a life as it was lived prior to dementia. Accordingly, it is the metaphysical relation that plays a decisive role in determining the ethical question of whether a person with dementia should or should not continue to live. For a critique of McMahan’s metaphysical approach to ethical questions, see Stephen Mulhall [19].

11 This is described by Guy Fletcher as the ‘shape of life hypothesis’ [21, p. 122].

12 Iris Murdoch’s husband John Bayley records her as saying during the progression of her dementia that she was ‘sailing into the dark’ [22].

13 Although a different discovery with different implications, Oedipus’s anguish when discovering he has killed his father and married his mother is a related kind of loss [23].

14 I note that an exception here could be cases where there is a profound change in a person’s moral qualities as a result of dementia. Nina Strohminger and Shaun Nichols draw on empirical findings indicating that family members do regard the personhood of a loved one with dementia as significantly altered when moral qualities change, far more so than with other changes such as memory loss and decreased cognitive function [24]. Such findings lead them to suggest that selfhood depends on moral traits more than on cognitive traits and capacities. In dementia, changes in what the authors describe as essential ‘moral traits’ of the self are far less common than other changes. And arguably, changes to former moral traits are a moral loss, but not a retroactive moral loss.

15 My use of the term ‘person-centred’ care is in reference to the influential approach developed by Tom Kitwood and Kathleen Bredin [25]. As a reviewer notes, professional carers do not have the experience of patients’ changing or having changed due to their dementia, as they typically do not know these patients prior to dementia onset, in contrast to family members and loved ones. But this care varies depending on the nature of the changes, as Strohminger and Nichols describe [24] (see footnote 14).

16 See Steve Matthews for a discussion of the link between previously loved music and the well-being of people living with dementia [27].
